# No evidence that within-group male relatedness reduces harm to females in *Drosophila*

**DOI:** 10.1002/ece3.1417

**Published:** 2015-01-31

**Authors:** Brian Hollis, Tadeusz J Kawecki, Laurent Keller

**Affiliations:** Department of Ecology and Evolution, University of LausanneBiophore, 1015, Lausanne, Switzerland

**Keywords:** *Drosophila*, inclusive fitness, kin selection, relatedness, sexual antagonism, sexual conflict, social evolution

## Abstract

Conflict between males and females over whether, when, and how often to mate often leads to the evolution of sexually antagonistic interactions that reduce female reproductive success. Because the offspring of relatives contribute to inclusive fitness, high relatedness between rival males might be expected to reduce competition and result in the evolution of reduced harm to females. A recent study investigated this possibility in *Drosophila melanogaster* and concluded that groups of brothers cause less harm to females than groups of unrelated males, attributing the effect to kin selection. That study did not control for the rearing environment of males, rendering the results impossible to interpret in the context of kin selection. Here, we conducted a similar experiment while manipulating whether males developed with kin prior to being placed with females. We found no difference between related and unrelated males in the harm caused to females when males were reared separately. In contrast, when related males developed and emerged together before the experiment, female reproductive output was higher. Our results show that relatedness among males is insufficient to reduce harm to females, while a shared rearing environment – resulting in males similar to or familiar with one another – is necessary to generate this pattern.

## Introduction

Sexual competition between males often leads to strategies that are harmful to females (Parker 1979; Arnqvist and Rowe 2005). For example, male *Drosophila melanogaster* harm females through persistent courtship and by transferring toxic seminal fluid proteins during mating (Chapman et al. 1995). Experimental elimination of male–male competition reduces this harm (Holland and Rice 1999; Crudgington et al. 2010) and increases the reproductive output of evolving populations (Maklakov et al. 2009; Hollis and Houle 2011).

Because the offspring of relatives contribute to inclusive fitness (Hamilton 1964), kin selection theory predicts that males should reduce direct competition with one another and levels of harm caused to females if competitors are genetically related to one another. A classic example of adjustment of sexual competitiveness that benefits females is “wife sharing” by related males in Tasmanian native hens (Smith and Ridpath 1972; Goldizen et al. 2000). However, such a relatedness-conditional strategy is only expected to evolve if two conditions are satisfied. First, males must be able to recognize kin, or at least assess the average relatedness of their competitors. Second, sexual competition must occur within groups which are both small enough to generate substantial variation in relatedness among males and are sufficiently stable on the timescale on which mechanisms of sexual competition act.

These conditions are difficult to satisfy in species with little social structure or territoriality. Therefore, the recent report (Carazo et al. 2014) that male *Drosophila melanogaster* reduce the harm inflicted on females when competing with kin is surprising. Carazo et al. (2014) found that female *D. melanogaster* kept with groups of brothers exhibited higher lifetime reproductive success than those kept with groups of unrelated males. They concluded that males adjust the intensity of intrasexual competition when competitors are brothers and this plastic response is likely a product of kin selection. This would be a highly interesting finding; despite numerous experimental tests there is little evidence that variation in within-group relatedness affects levels of cooperation and competition in insects (Keller 1997; Breed 2014). Unfortunately, in the experiments of Carazo et al. (2014), relatedness was confounded with differences in rearing between treatments: related brothers developed together in the same vial and had an opportunity to interact with one another as larvae and adults, while unrelated males were reared separately and had no experience with one another. The roles of relatedness and the shared rearing environment in generating these results are therefore impossible to disentangle.

Here, we show that relatedness among males is insufficient to generate differences in female lifetime reproductive success, while the shared rearing environment is necessary. In our experiment, females exposed to three males raised separately had the same reproductive success irrespective of whether the males were brothers or unrelated. In contrast, females housed with related males reared in a shared vial showed the highest reproductive success. We conclude that this phenomenon is unlikely to reflect a strategy ultimately driven by kin selection and propose a more parsimonious explanation for the results of both our experiment and that of Carazo et al. (2014): males reared together are similar to one another or familiar with one another, and this influences the behavior of males or females in a way that increases their direct fitness.

## Methods

### Experimental flies

Flies used in all experiments came from a long-term laboratory adapted population (the IV population) that was initiated from *D. melanogaster* collected in 1975 (Charlesworth and Charlesworth 1985). All flies were reared and maintained as adults in vials with standard 2% yeast food (water, agar, brewer's yeast, cornmeal, sucrose, and Nipagin [Sigma-Aldrich, Buchs, Switzerland]) on a 12L:12D cycle.

### Manipulation of relatedness and rearing environment

To generate experimental male triplets, eggs from 24-h pairings of 1-week old virgin males and females were divided equally between three rearing vials. These crosses were used to set-up three mating treatments: (1) three virgin brothers collected from one randomly chosen vial (*related-familiar* treatment); (2) one virgin brother collected from each of the three different vials of a cross (*related-unfamiliar* treatment); or (3) one virgin male collected from a randomly chosen vial from three different parental crosses (*unrelated-unfamiliar* treatment). No parental crosses were re-used, so offspring originating from a given parental pair were only used in one experimental replicate and in a single treatment.

Virgin females were collected from an independent set of crosses (10 bottles in total, each with 20 males and 20 females). Male triplets were placed with an unrelated, same-aged, virgin female 48 h after being composed. All flies were transferred to new vials every 3 days until female death or cessation of reproduction. Every 9 days, male triplets were replaced with a new triplet identical in composition (from the same cross and of the same age as the original triplet). In order to accomplish this, parental pairs were kept at 18°C for 8–9 days without live yeast while not producing experimental triplets. They were then placed in a fresh vial at 25°C with live yeast to encourage egg-laying, and the previous procedure for producing triplets was repeated. Replicates for which parental pairs failed to generate sufficient eggs on the second crossing were discarded, as were those where experimental females failed to produce a first brood, yielding *n* = 22–25 females per treatment. All emerging flies from all broods of each female were collected until no further adults emerged. The age of last reproduction and age of death were also recorded for each female. The experiment ended after 24 days, at which point all females had ceased reproducing.

### Statistical analysis

Statistical analysis was performed in SAS 9.2 (2011) using linear models, linear mixed models, and proportional hazards regression. Specific contrasts of interest were constructed within each model framework. Total reproductive output was modeled in PROC GLM with treatment (related-familiar, related-unfamiliar, and unrelated-unfamiliar) as the only effect. Female reproductive output over time was modeled in PROC MIXED with treatment as a fixed effect and the identity of each female included as a random effect to account for repeated measurements. For age of death and age of last reproduction, we used two different approaches. First, we modeled each in PROC GLM with treatment as the only effect, assigning a maximum value of 24 days for the few females still alive at the end of the experiment. Next, we used Cox proportional hazards regression (PROC PHREG), accounting for this right-censored data, in additional analyses testing the same main effect of treatment. Both approaches yielded the same conclusions – we report both in the text and plot simple means.

## Results

### Female reproductive success

**Figure 1:**
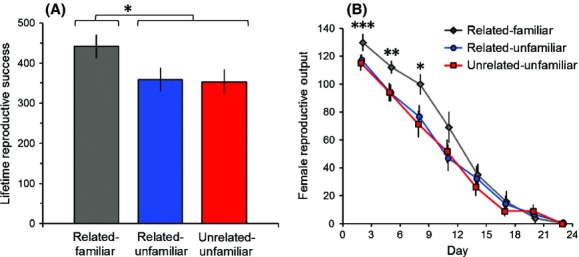
Lifetime reproductive success (mean ± SE) was higher for females housed with brothers reared together (related-familiar) than those housed with related males that were not reared together (related-unfamiliar) and unrelated males that were not reared together (unrelated-unfamiliar) (A). The advantage in reproductive success of females housed with brothers over females from both other treatments arose immediately and lasted through the first three broods (B). ***P <* *0.001, **P <* *0.01, *P <* *0.05.

Lifetime reproductive output (the sum of all offspring across all broods) did not differ between females housed with brothers reared apart and females housed with unrelated males reared apart (Fig.1A, related-unfamiliar vs. unrelated-unfamiliar: *F*_1,65_ = 0.02, *P* = 0.89). By contrast, females that were housed with males reared together produced 23–25% more offspring than females housed with males reared apart (Fig.1A, related-familiar vs. related-unfamiliar and unrelated-unfamiliar: *F*_1,65_ = 5.78, *P* = 0.02).

Examining reproduction over time reveals that the advantage of females housed with brothers reared together arises immediately. Reproductive output was higher in females housed with brothers reared together than in the other treatments for the first three broods of offspring (Fig.1B, male treatment effect: *F*_1,65_ = 13.28; related-familiar vs. related-unfamiliar and unrelated-unfamiliar, brood 1: *F*_1,65_ = 13.28, *P* < 0.001; brood 2: *F*_1,65_ = 10.53, *P* < 0.01; brood 3: *F*_1,65_ = 6.87, *P* = 0.01). Reproductive output declined over time for females from all treatments (Fig.1B, brood effect: *F*_1,325_ = 1012.84, *P* < 0.0001). Because of this difference between treatments in the number of offspring in the first three broods, this decline was significantly steeper in females housed with brothers reared together (Fig.1B, treatment × brood interaction: *F*_2,325_ = 5.53, *P* < 0.01; slope contrast, related-familiar vs. related-unfamiliar and unrelated-unfamiliar: *F*_1,325_ = 10.89, *P* < 0.01).

### Female age of death and last reproduction

**Figure 2:**
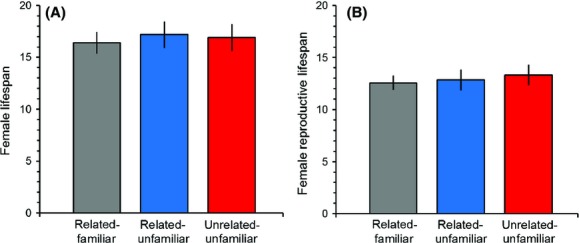
Female lifespan (mean ± SE) (A) and the age of last reproduction (mean ± SE) (B) did not differ between females from any of the treatments.

Female lifespan did not differ between any experimental treatments (Fig.2A, linear model treatment effect: *F*_2,65_ = 0.05, *P* = 0.95; proportional hazards model treatment effect: Wald *χ*^2^ = 0.27, *P* = 0.87). Females ceased reproduction on average several days before death and this did not differ across treatments (Fig.2B, linear model treatment effect: *F*_2,65_ = 0.19, *P* = 0.83; proportional hazards model treatment effect: Wald *χ*^2^ = 0.5911, p = 0.74).

## Discussion

Recent work testing predictions of kin selection theory suggested that male relatedness can modulate intrasexual competition and reduce harm to females (Carazo et al. 2014). Our study finds no difference in lifetime reproductive success between females exposed to related males and those exposed to unrelated males when all experimental males are reared separately. Conversely, females kept with brothers who shared a rearing vial and had experience with one another had the highest lifetime reproductive success. This advantage emerged immediately and lasted for over 1 week, a period which accounted for the majority of female lifetime reproduction. These results thus show that relatedness among males is insufficient to reduce female harm and demonstrate that shared rearing environment, resulting in nongenetic similarity of males or their familiarity with one another, is necessary to reduce female harm.

While we find no evidence for relatedness as the proximate factor mediating this response, it would still be possible for kin selection to play a role. However, this seems unlikely for two major reasons. First, even in social insect species where relatedness is known to play a key role in regulating social behavior, previous research has shown that individuals usually do not discriminate between kin classes within their colony (Keller 1997; Breed 2014). Second, *Drosophila* populations are unlikely to be sufficiently viscous to generate a reliable association between relatedness and familiarity. Females lay eggs one by one, moving around between successive ovipositions (Yang et al. 2008), and suitable oviposition sites attract multiple females. Moreover, larvae do not form family groups and often crawl considerable distances across the food patch (Osborne et al. 1997). Upon completion of feeding, the larvae move away from food to pupate (Sokolowski 1985; Medina-Munoz and Godoy-Herrera 2005), thus further reducing the likelihood that the adults males encounter in their adult life are siblings rather than random members of the population. *Drosophila* ecology and life history therefore suggest familiarity is unlikely to act as a reliable proxy for relatedness. An additional experimental treatment with unrelated but familiar males is not possible for technical reasons in our design, but could confirm a role for male relatedness if females in this treatment did not show elevated reproductive output. The opposite result – females showing elevated reproductive output when housed with unrelated but familiar males – would not rule out an ultimate explanation rooted in kin selection, however, if familiarity did in fact serve as a necessary signal of relatedness.

Importantly, our results and those of Carazo et al. (2014) can be parsimoniously explained without invoking kin selection, because there are many ways by which a shared rearing environment might affect the level of harm inflicted by males. First, it is possible that familiarity originating during development or in the few hours following emergence, before experimental triplets were formed, resulted in reduced aggression and therefore less intense competition that translated to reduced harm. Familiarity is known to be important for aggressive behavior and hierarchy formation – male flies remember individuals they have encountered and reduce aggression toward these familiar males (Yurkovic et al. 2006). Further, a single social defeat can turn strains that are normally winners of aggressive encounters into losers in future encounters (Penn et al. 2010). It is for these reasons that studies of aggression in *Drosophila melanogaster* often isolate males for several days before trials (Chen et al. 2002), or even separate pupae before adults eclose (Penn et al. 2010), in addition to using individuals that were not raised together (Dierick 2007), in order to exclude early life experiences. Direct familiarity (past experience with a certain individual) or phenotypic familiarity (past experience with a similar individual) are also known to influence both male and female mate choice (Tan et al. 2013).

Another possibility is that increased phenotypic similarity between males reared together modifies male or female behavior. Females are known to favor rare males (Spiess 1982), for example, so it is not hard to imagine differences in choosiness or resistance that hinge on the similarity of males. Indeed, there is more mating in heterogeneous groups than in homogeneous groups (Krupp et al. 2008), and female reproductive output depends on the heterogeneity of groups of males (Billeter et al. 2012). This kind of similarity would show up in male pheromone (cuticular hydrocarbon or CHC) profiles, which are sensitive to social environment in a genotype-dependent manner (Kent et al. 2008). Flies with similar CHC profiles could be generated by shared housing (Farine et al. 2012), so that males reared together are likely to smell more like one another than males reared separately. Females can distinguish between males from the same strain that were reared in different bottles, likely due to stochastic differences in odor from one culture to the next (Hay 1972), so it may simply be that female mating behavior changes when competing males originate from the same vial. Further, males invest more in sperm in the presence of rivals, who are identified by these same olfactory cues (Garbaczewska et al. 2013).

Whatever the exact mechanism at work, our experiment unambiguously demonstrates that the shared rearing environment is necessary to inhibit male competition and female harm in *Drosophila*. The idea that levels of kinship might modulate harm to females (Pizzari and Gardner 2012) is an appealing one, but it is impossible to reach this conclusion without experiments that either manipulate relatedness independently of other factors or explicitly measure direct and indirect fitness benefits.
